# Supplementary material for: Inhibition of Bromodomain Proteins Enhances Oncolytic HAdVC5 Replication and Efficacy in Pancreatic Ductal Adenocarcinoma (PDAC) Models
Source: Int J Mol Sci. 2024 Jan 19;25(2):1265. doi: 10.3390/ijms25021265 (PMC10816486; doi:10.3390/ijms25021265)
Supplement: Supplementary file 1 [file ijms-25-01265-s001.zip › ijms-2730020-supplementary.pdf]

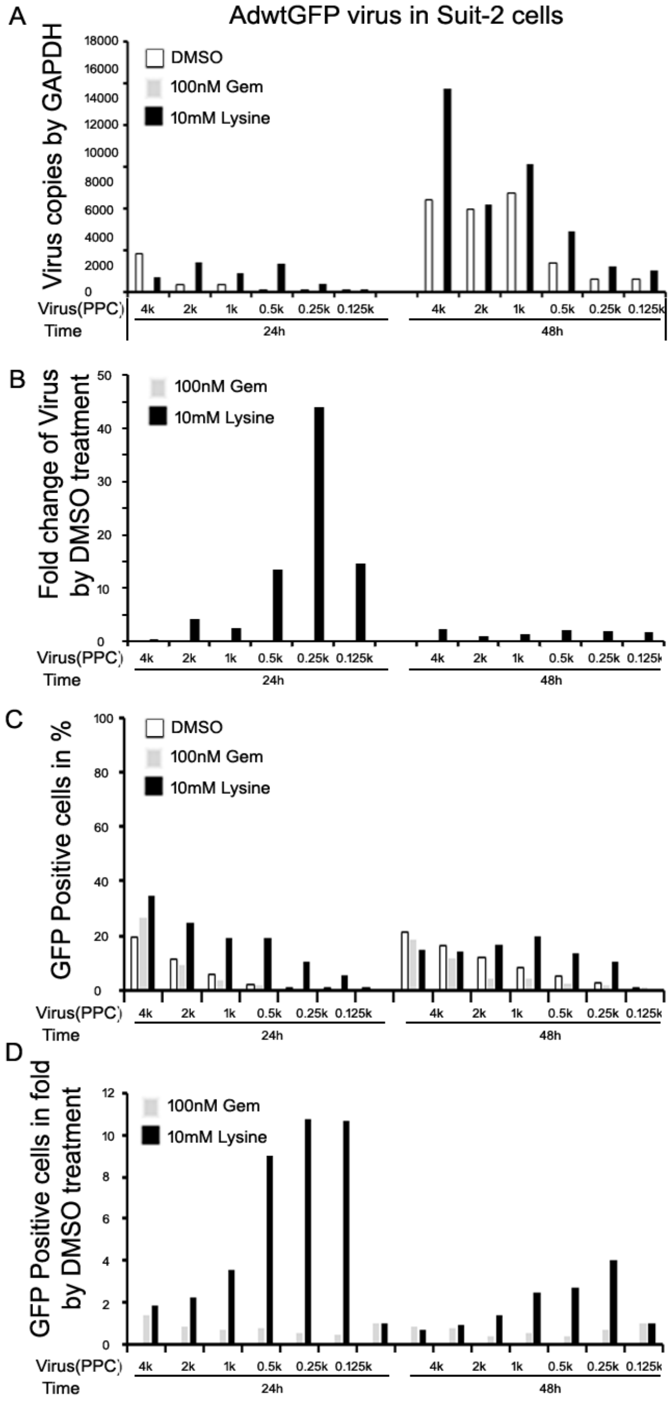

Figure S1. Optimization of conditions for high-throughput screening (HTS). Suit-2 cells were seeded in 384-wells and infected with Ad5wtGFP at increasing doses and time. Expression of GFP was used as a marker for viral uptake (early gene expression) and replication in the presence of test compounds known to increase and decrease viral uptake and replication, Lysine (10mM) and Gemcitabine (100nM), respectively. **A)** Viral DNA amplification by qPCR in cells infected with increasing doses of Adwt GFP (125-4000ppc) and analyzed after 24 and 48h. **B)** Fold change of viral DNA amplification compared to untreated infected cells. **C)** Infected cells (% of untreated infected cells). **D)** Fold increase in viral infected cells, treated compared to untreated infected cells. These data proved that the experimental conditions supported detection of changes in viral uptake and replication and were suitable for HTS of the compound library.

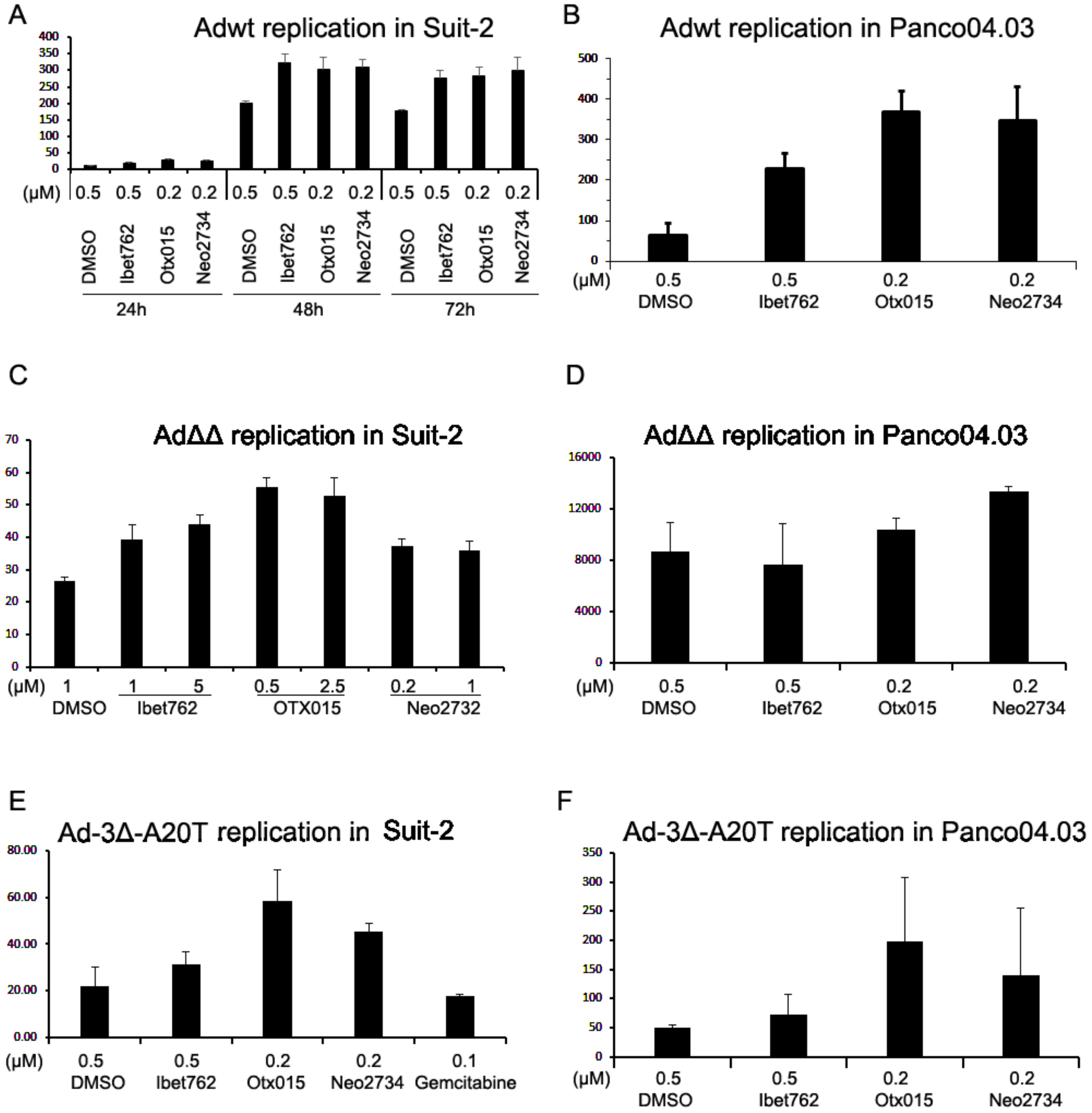

Figure S2. Replication of Adwt, AdΔΔ and Ad-3Δ-A20T in Suit-2 and Panc04.03 cells determined by TCID50. A) Increased Adwt replication in combination with iBET-762 (0.5μM), OTX-015 (0.2μM) and Neo-2734 (0.2μM) compared to control infected Suit-2 cells. Media and cells were harvested at 24, 48 and 72h after infection. **B)** Adwt replication in Panc04.03 cells, analysed after 72h. **C)** AdΔΔ replication in Suit-2 cells 48h post infection. **D)** AdΔΔ replication in Panc04.03 cells 48h post infection. **E)** Ad-3Δ-A20T replication in Suit-2 cells 48h post infection. **F)** Ad-3Δ-A20T replication in Panc04.03 cells 48h post infection.

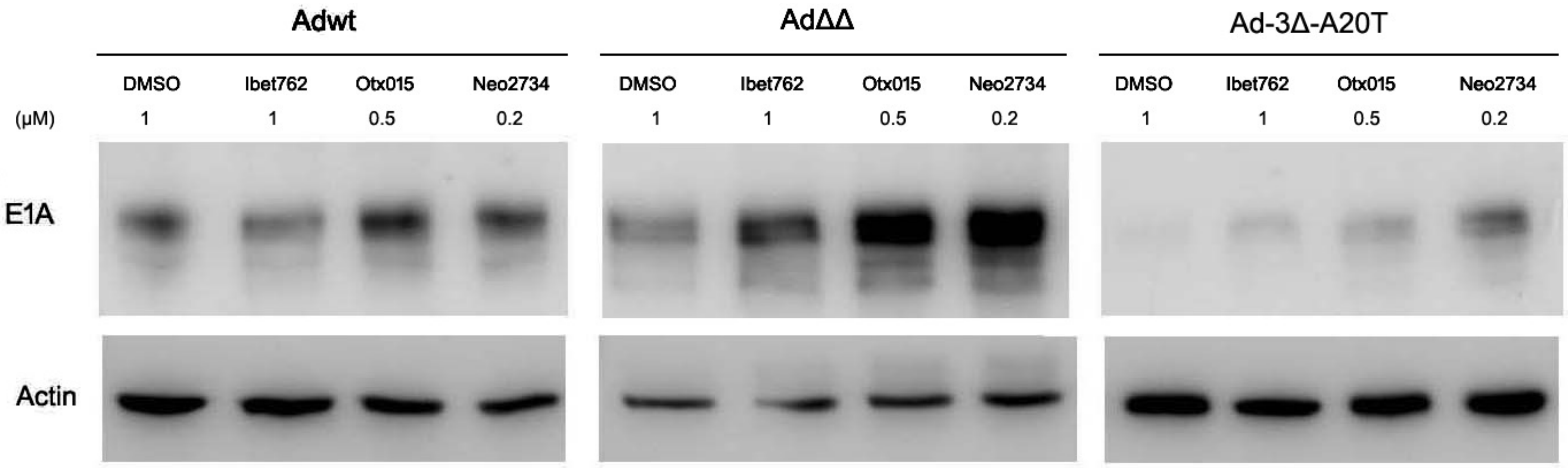

Figure S3. **Immunoblotting of the viral E1A protein.** Suit-2 cells were infected with Adwt, AdΔΔ or Ad-3Δ-A20T and harvested for E1A expression 48h post infection and drug treatment. Representative blots.

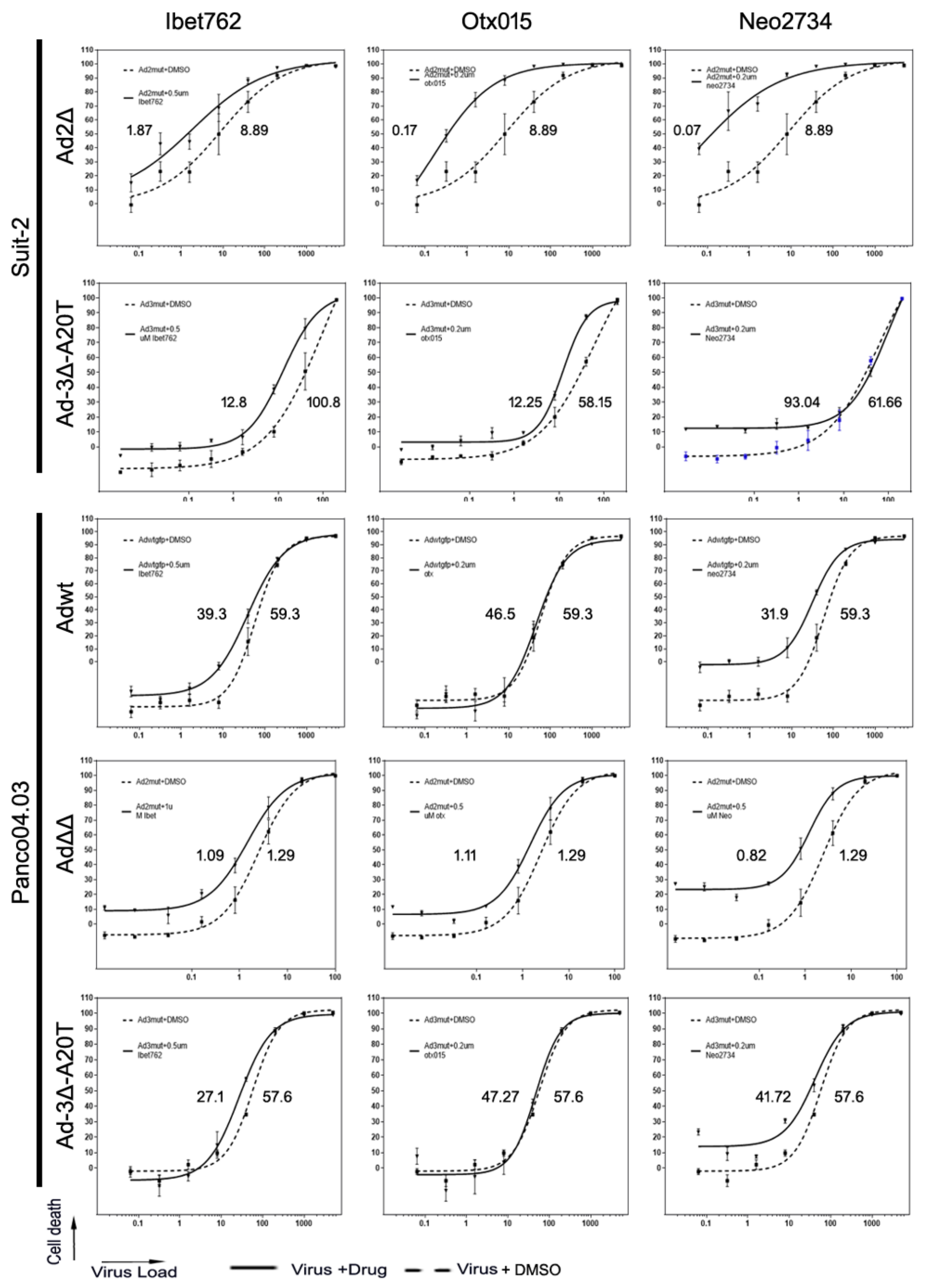

Figure S4. The BRD4 inhibitors enhance cell killing in oncolytic virus infected PDAC cell lines. Upper panels; Suit-2 cells were infected with serial dilutions of AdΔΔ followed by addition of iBET-762 (0.5μM), OTX-015 (0.2μM) and Neo-2734 (0.2μM). Cell viability was determined 6d after treatment/infection (MTS assays). Suit-2 cells were infected with serial dilutions of Ad-3Δ-A20T followed by addition of the inhibitors and cell viability assays, as described above. Lower panels; Panc04.03 cells were infected with serial dilutions of Adwt or AdΔΔ followed by addition of the inhibitors and cell viability assays, as described above. Panc04.03 cells were infected with serial dilutions of Ad-3Δ-A20T followed by addition of the inhibitors and cell viability assays, as described above.

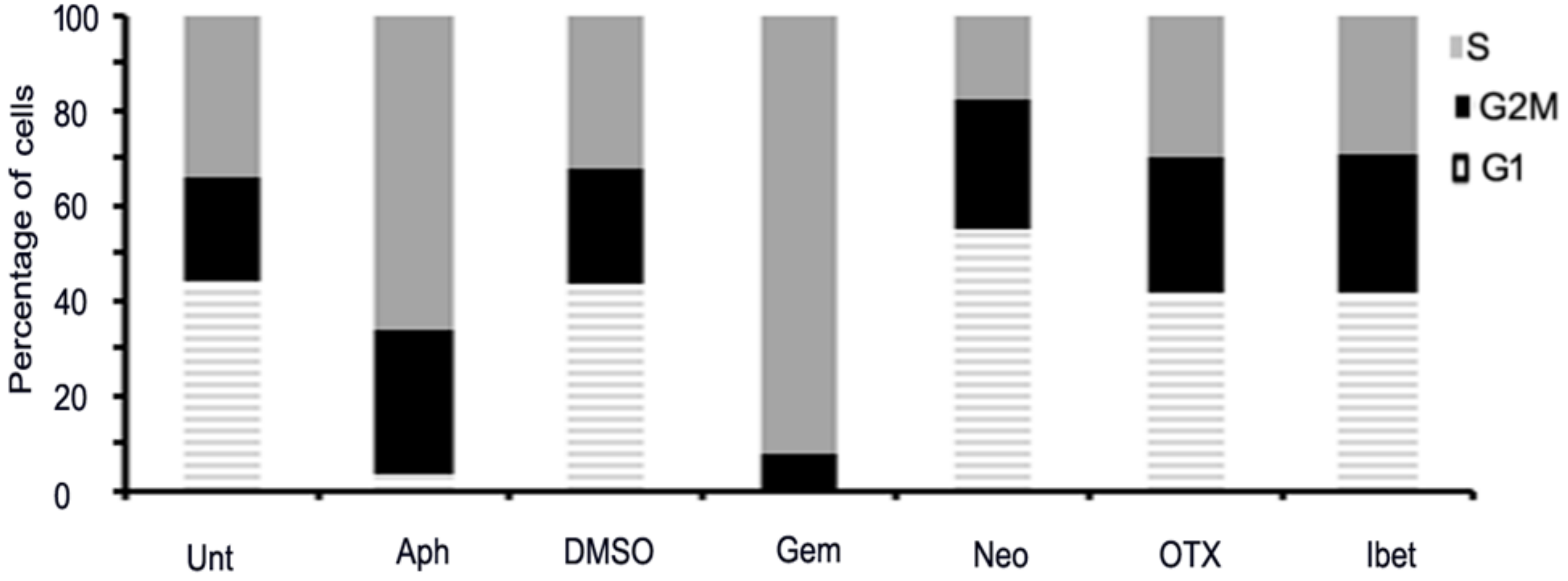

Figure S5. The inhibitors attenuate cell cycle progression and proliferation. Suit-2 cells were synchronized with aphidicolin (0.2µM) in serum-free DMEM for 16h, followed by addition of the inhibitors iBET-762 (0.5µM), OTX-015 (0.2µM) and Neo-2732 (0.2µM) for 24h. Cells were analysed by flow cytometry to determine changes in DNA content and cell cycle progression

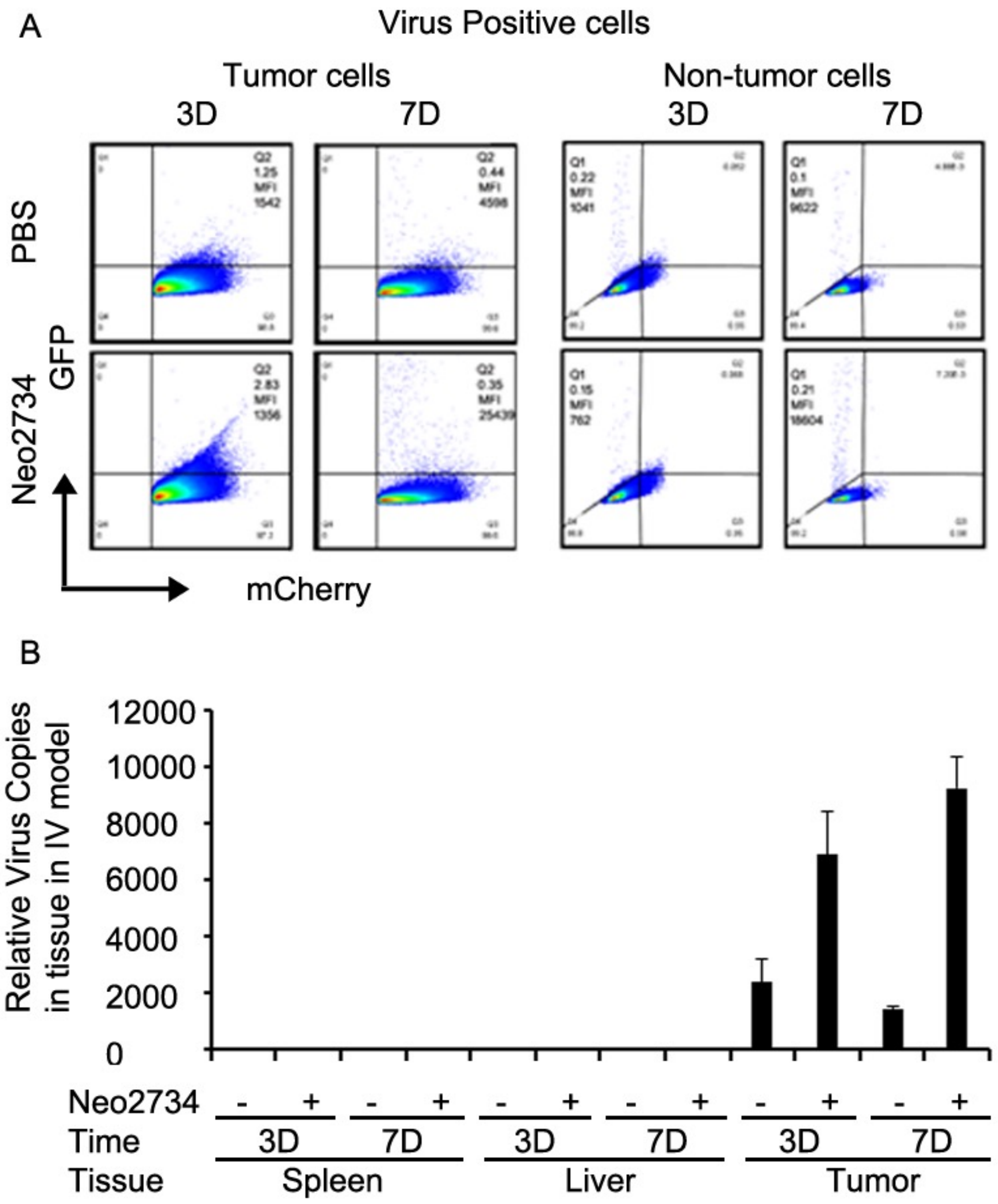

Figure S6. Neo-2734 selectively increases viral replication and distribution in Suit-2 subcutaneous xenografts *in vivo* but not in non-tumour tissue. Suit-2 cells (5x10^{6}) expressing mCherry were implanted subcutaneously in Matrigel (1:1) in NOD2SCID mice. Ad5wtGFP virus (1x10^{10}vp/dose) was administered intravenously when tumours were 100±20mm^{3}. Neo-2734 (1µg/µl) or PBS was administered intraperitoneally. **A)** Animals were culled at day 3 (3D; Left panels for each tumour and non-tumour) and day 7 (7D; Right panels for each tumour and non-tumour) after infection/treatment and tumours, liver and spleen were harvested and processed for flow cytometry analysis. **B)** Virus replication analysed by qPCR with E2A primers in tumour cells and non-tumour cells (spleen and liver) harvested 3d and 7d after treatment, n=3/group.

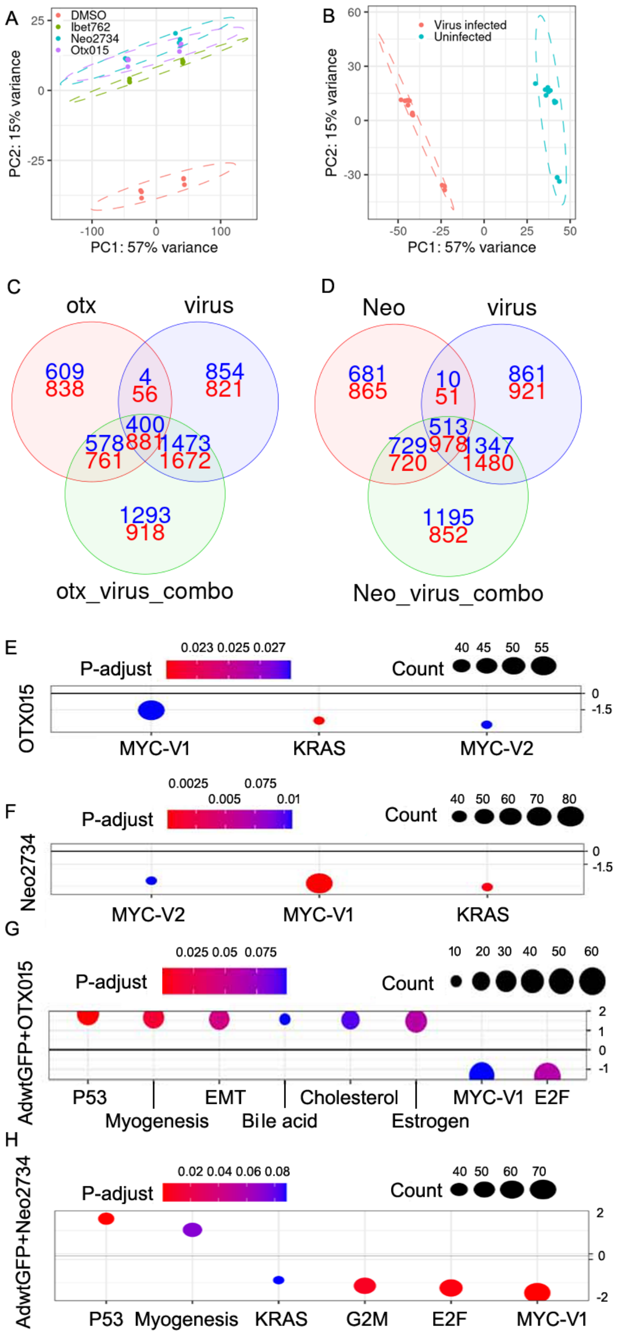

Figure S7. Differential regulation of genes and signalling pathways by drugs and virus. RNA sequencing was carried out following single agent and combination treated Suit-2 cells. The effect of either drugs **(A)** or virus **(B)** on the transcriptome landscape was graphed by variances (PCA plots) compared to control treated cells (DMSO). **C-D)** Genes regulated by virus or OTX-015 **(C)** or Neo-2734 **(D)** were numbered and compared to the respective combination treated cells. **E-H)** The RNA-seq data from Fig. 6 was analysed by GSEA using Broad Institute Hallmark gene sets (62). Normalised enrichment scores and Benjamini-Hochberg adjusted p-values are shown. Positive normalised enrichment scores indicate upregulated pathways while negative scores denote that the pathway is downregulated by treatment. The top pathways affected by either drugs **(E, F)** or in combination with virus **(G, H)** are listed.

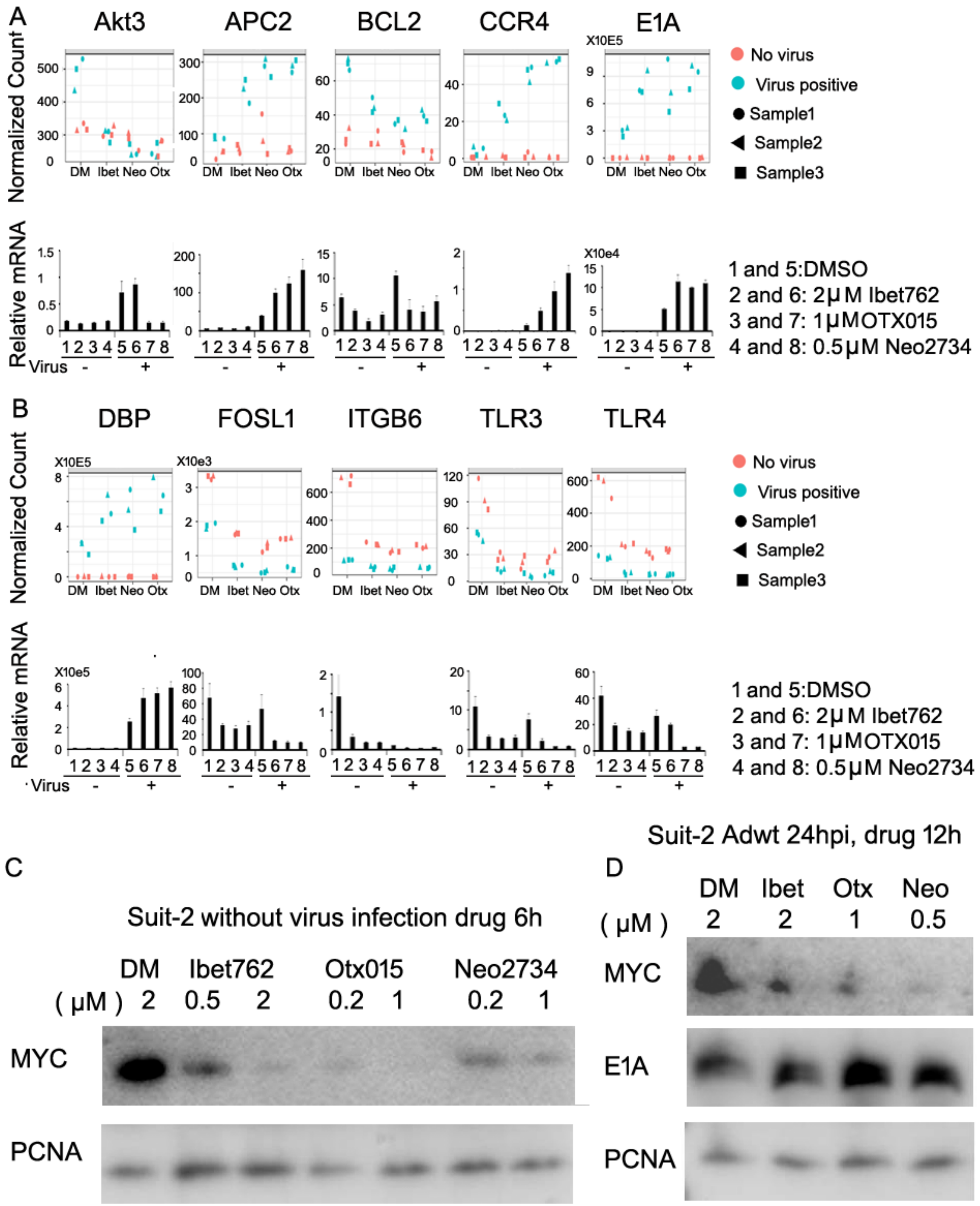

Figure S8. Global effects of Ad5wtGFP and bromodomain inhibitors on viral and cellular transcriptomes. Validation of representative genes regulated by either virus or inhibitor in RNA sequencing and confirmation RT-qPCR. **A-B)** The PDAC Suit-2 cells were infected or uninfected by Ad5wtGFP for 12h, and treated with the inhibitors for another 12h. GFP positive cells were isolated by fluorescence activated cell sorting. Total RNA was extracted and subjected to RNA sequencing in biological triplicate samples for each treatment. Main changes are the increased expression of the viral DBP and E1A proteins and the decreased levels of αvβ6-integrin and TLR3 and TLR4. **C, D)** Decreased expression of c-Myc in the presence of the inhibitors alone and in combination with Adwt despite the high levels of expression in control treated and virus infected only Suit-2 cells.
